# Two Clinical Cases

**Published:** 1868-04-01

**Authors:** Tom O. Edwards

**Affiliations:** Lancaster, Ohio


### TWO CLINICAL CASES.

BY TOM O. EDWARDS, M.D., LANCASTER, OHIO.

*Professor :*

I was called two weeks since, by request of Dr. Dawson, physician in attendance, to see a Mrs. W., four miles from our city, aged 78 years. The Doctor could not visit her that day, and requested I should be sent for. I was prepared to see a case of stubbornness, and went armed. You know I have great faith in the therapeutic application or use of remedies.

The controlling indication, nausea and vomiting, had existed nearly a month, with slight remissions. The patient told me for more than twenty years "she had had such spells; sometimes the doctor could, and sometimes he could not, help her. This was the longest and hardest; and, for the first time in her life, she believed she would now die." She was emaciated; had retained nothing on the stomach for two weeks, except one night mush and buttermilk, which was ejected unchanged in the morning.

I remained with her all the afternoon; used all the appliances of the art, through the stomach, hyperdomic, per enema; and finding all not available, induced anæsthesia, and directed its continuance. Met Dr. Lawson in the morning; then got such a history of suffering as I will not detail. During the night, three or four large discharges of an inodorous matter had passed the bowels, and the vessel emptied on the snow in the yard. We examined these, and found the "prune juice" so much talked of in scirrhous or cancerous stomach. Looking over the snow, we saw some fifty places on which the contents of the basins had been poured after vomiting, and all had the green color of bile; and the Doctor told me she never had vomited unless there was more or less of this green matter with the ejections, and in his life he never saw a case characterized by this billiousness. He had gone the round, and found, if any thing had ever acted medicinally (and this he doubted), it was *Sub. nit. of bismuthum.* The case had given him much trouble, for more than five years. He had had various and conflicting opinions, at different times, of its pathology; but at no time, such as were entirely satisfactory. He is not easily conquered; but in this case, confessed there was something neither his books nor his observation satisfied him of its cause or origin.

I stated three or four large dejections occurred the night before, accompanied with large flatulent discharges. These produced subsidence of the abdominal walls; and then, for the first time, we found a tumor — that over or near the cardiac orifice; and then schirrus of cardiac orifice was diagnosed. This, you know, is rare in such affections of stomach; the tumor, in ninety per cent, is found in pyloric orifice.

That afternoon, our patient died — calmly, and with no more suffering than had existed for four or five weeks.

Two days after, we were allowed to "examine only the stomach." You know the prejudice, and as we asked only to open the stomach, after a long time we got the consent of friends, and particularly as the patient had requested it, the objections were removed.

On going to the house with Dr. Geo. W. Brewster, I predicted we should find gall stones in the gall bladder; and truly it was so. The tumor we felt was the contents of the gall bladder, which, on being opened, I counted, and put in Dr. Dawson's hands. One hundred and nine gall stones were taken from the gall bladder, and after the removal of the corpse, one more was found on the floor, making one hundred and ten. Again, no man could have placed them again in the bladder. They were of all imaginable shapes, from the size of a grain of corn to ten times that size; filling, dovetailing, every space in the bladder; and one, the largest, in the duct, closing it so entirely as that it could neither be pressed forward nor backward. The duct, from the conjunction of the hepatic (?) duct with the duct from the bladder, and they forming the *ductus communis choledochus*, was open; and thus the bile, secreted as excreted, ran into the natural opening of the intestine. The stones were pearly white, soft, friable, and greasy to the feel. This number was remarkable, and the curving of their formation and deposition, more remarkable. I have a number, and will send them if you desire. Had I all of them, I would forward for your cabinet, as a curiosity. *There was also schirrus in pyloric orifice.*

After a correspondence with several prominent men in the profession, I deem it my duty to report the following case. In my own practice it was new, and I could find nothing in the various books similar to it; but still I feared it might be one of "Charley's discoveries," known to every body else. I thus communicated with distinguished professors; it was new to them.

I was called, on the morning of Dec. 7th, to see a Mrs. B., in our city. I was told by her husband that Dr. Boerstler had been with her all night; and, being very feeble and unwell, sought my assistance. She was the mother of six children; had some trouble in previous labors, but never such as then existed. Dr. B. stated he was called the evening previously; touched, but could not satisfactorily make out the presentation; and, as the pains were slow, went to bed, desiring to be called when wanted. Was called at 2 A. M., and found the labor active, but no progress; one hand presenting, and nothing more. He, finding the hand very diminutive, indicating a premature birth, pulled — as he thought, very gently — and found the arm separated from the shoulder; and looking into the vessel, under the bed, I saw the arm of an immature child. The doctor was lying upon a couch, and, as the room was very cold, was covered with blankets. He requested me to make an examination, and to take charge of the case, as he was exhausted and ill. On introducing my hand, I found the os dilated; I passed my entire hand *to the middle of the uterus*, and found a second arm presenting, so firmly girt about by an hour-glass contraction of the uterus as to preclude the passage of a finger. The slightest traction on the hand, released it from the shoulder; and the space occupied by the arm was closed, as with a drawn string, and resisted dilatation. The patient was excited, cold, and restless; demanding immediate means of relief from what she described as a pain such as a hot iron would be supposed to give at the seat of contraction. She had taken some brandy. Circulation feeble, and her importunities great for relief from this pain. I gave her a prompt anodyne of fluid extract *Tr. opii*, *deodorata*; sent for four ounces of fluid extract *Belladonna;* injected over the uterus, hypodermically, a portion of the extract, and applied a cloth, wet with the extract, over the entire abdomen; pinned up and covered the patient with blankets, got the room warm, and awaited results. Our patient slept soundly, three hours; awakened refreshed, and commenced her importunity for relief. She was fully advised of the trouble, and as her stomach was becoming irritable, it was decided to use additional *Tr. opii*, per enema; and as we were preparing the instrument, a violent pain came on, and on touching, *the child's breech presented;* three pains completed the delivery of child and secundines; the uterus contracted, and "she was as safe as if she was in church,'" and had an unusually good "getting up."

This was evidently a case of hour-glass contraction in labor, preventing the expulsion of the child; and as such, was unique. So say my corrrespondents; but all have had occasional cases of such contraction, following labor, and retaining placenta; — none such as this.

The statement of our patient, an unusually intelligent and observing lady, was, that she did not come to the full period of gestation; and that for four or five months she felt as if there was a contraction or hardening of the uterus — *a burning, severe, constant pain*, which no position would benefit, nor any anodynes entirely dispel; and she was prepared for some abnormal result in her labor; and, placing her fingers on the abdomen, clearly pointed out the place of the contraction as the seat of her constant pain.

Dr. Boustler has practiced more than half a century, has a large obstetric practice, and never saw such a case; and in my own practice of rising thirty years, it never before occurred; and I give it as a rare case, and also to show the advantage of patience and trusting to nature, as a general rule.

I used the *Belladonna* analogically: if it will dilate the os, why not dilate a spasm of circular fibres?

